# A meta-analysis of the effect of physical activity programs on fundamental movement skills in 3–7-year-old children

**DOI:** 10.3389/fpubh.2024.1489141

**Published:** 2025-01-07

**Authors:** Yunjiao Yang, Xiaojin Mao, Wenhao Li, Botian Wang, Lixia Fan

**Affiliations:** Department of Physical Education, Shandong Normal University, Jinan, China

**Keywords:** children, fundamental movement skills, physical activity programs, locomotion skill, object control ability, stability, meta-analysis

## Abstract

**Introduction:**

This study aimed to systematically review the effects of different physical activity programs on the fundamental movement skills of 3 - 7-year-old children.

**Methods:**

For this review, the databases of CNKI, Web of Science, and PubMed were searched to collect relevant literature on the effects of different physical activity program interventions on fundamental movement skills, and a total of 10 articles with 1,121 subjects were included. The Cochrane Risk of Bias Assessment Tool was used to assess the quality of the literature, and meta-analysis was performed using Review Manager 5.4 software.

**Results:**

Physical activity significantly influenced children’s running ability, horizontal jump, dribbling the ball, kicking ability, catching ability, overhand throwing, striking a stationary ball, and dynamic balance. However, the intervention effect was insignificant for the hop and underhand throwing abilities. The intervention effects for running ability, horizontal jump, kicking ability, and catching ability were better at less than 12 weeks than at 12 weeks and above. In addition, an intervention duration of 90 min or more was better than less than 90 min for running ability and horizontal jump.

**Conclusion:**

Future research is recommended to focus on the common factors of the intervention effects of physical activity programs to develop more precise and effective intervention practices to further improve children’s fundamental movement skill levels.

## 1 Introduction

Fundamental Movement Skills (FMS) refer to the ability to coordinate basic human movements (1). They are considered to be the ‘building blocks’ of more complex movement skills required for games or other physical activities (2), including mainly gross motor skills such as running, jumping, etc., object control skills such as grasping and throwing a ball, and stability skills such as balancing and swinging (3). According to Gallahue, a model of movement development was proposed in 2012, broadly divided into four phases: Reflexive Movement Phase, Rudimentary Movement Phase, Fundamental Movement Phase, and Specialized Movement Phase. The Fundamental Movement Phase is from 2 to 7 years old and covers toddlers and early childhood, as children gradually develop more complex movement skills and coordination during this period, including fundamental movement skills such as running, jumping, throwing, and so on (4). As a result, during this stage, children’s coordination and object control abilities improve significantly. Adequate exercise practice and effective instruction are needed to allow children to increase their confidence in exercise and their FMS levels. Children with high levels of FMS will be more able to participate in physical activity (5), improve cardiorespiratory fitness (6), and reduce obesity rates. Children with high levels of FMS will be able to participate in a wide variety of physical activities with high levels of FMS (7).

Physical activity (PA), which encompasses all voluntary bodily movements produced by skeletal muscles that result in energy expenditure (8), is an important factor in promoting healthy child development, and participation in physical activity in early childhood confers considerable benefits, such as improving obesity, promoting cognitive development, and skeletal, psychosocial, and cardiometabolic metabolism (9). The World Health Organization (WHO) recommends that preschool children should engage in at least 60 min of moderate to vigorous physical activity (MVPA) per day (10), yet more than 85% of children and adolescents worldwide do not meet the WHO guidelines. With continuous social and economic development, people’s living standards continue to improve. The increase in sedentary behavior and lack of physical activity has led to an increase in the risk rate of obesity in children year by year (11, 12), and physically insufficient behaviors are easy to form in childhood and continue into adulthood, which ultimately leads to other health problems (13). Therefore, it is essential to understand the underlying mechanisms of physical activity (14).

Stodden et al. noted that PA and FMS have a mutually beneficial relationship, with PA promoting the development of FMS during childhood and FMS promoting PA involvement at a slightly older age (15). One study confirmed the positive correlation between FMS and organized PA (16). Another narrative review found that 8 out of 11 studies on children aged 3–5 years showed a significant relationship between FMS and PA (17). However, longitudinal studies have highlighted significant uncertainty in directly linking physical activity to FMS (18). In a meta-analysis, researchers found that the association between FMS and PA was inconsistent in children (3). Another investigation of studies that used PA as a predictor of FMS found that objective measures of moderate to vigorous physical activity (MVPA) at 3.5 years of age did not predict FMS in 5-year-old Australian children (19). This suggests that our understanding of how physical activity impacts FMS is still incomplete, and further research is necessary.

Notably, children’s FMS does not develop naturally over time (20) but requires intentional cultivation through various interventions, such as ongoing guidance, feedback, and practice (21). Consequently, a range of physical activity programs tailored to children’s physical and mental developmental stages has been created, aiming to enhance FMS. Current research has indicated that jumping classes (22, 23), rhythmic classes (24), active play (25), core movement instruction (26), strength training classes (27), motor intervention classes (28, 29), and ball games (30, 31) are all beneficial in developing children’s FMS. These programs promote the comprehensive and balanced development of children’s FMS by integrating diverse sports elements and employing open-ended physical education games that emphasize enjoyment and variety (32, 33). The main purpose of this study is to explore the effects of different types of physical activity programs on improving children’s FMS levels. The study focuses on running ability, jumping ability, object control ability, and balance ability as key indicators. Running and jumping directly reflect children’s coordination and lower limb strength, which are core skills in daily life and many physical activities. Object control skills include throwing and catching, reflecting children’s upper body coordination and fine motor skills. Balance skills help children maintain stability in both static and dynamic situations, reduce the risk of falling, and improve overall body coordination. By using meta-analysis to assess the impact of physical activity on children’s FMS, the study aimed to elucidate a precise perspective on how physical activity interventions can be more effective in improving children’s levels of fundamental movement skills, laying the groundwork for more effective promotion of lifelong physical education in the future.

## 2 Materials and methods

### 2.1 Experimental approach to the problem

This study was guided by the Preferred Reporting Items for Systematic Evaluation and Meta-Analysis (PRISMA) (34), registered in the PROSPERO database under CRD42024548531. By searching the databases of CNKI, PubMed, and Web of Science, the search formula in Chinese and English was (Fundamental movement skill OR gross motor movement) AND (Physical activity OR exercise) AND (children OR kid) AND (RCT OR Randomized controlled experiment); the search formula in English was (Fundamental movement skill OR gross motor skill) AND (physical activity OR Physical exercise) AND (children OR kid) AND (RCT OR Randomized controlled experiment).

### 2.2 Eligibility criteria

#### 2.2.1 Subjects for inclusion

(1) Randomized controlled trials of different physical activity programs that intervene in children’s basic movement skills and language restricted to Chinese and English. (2) Children aged 3–7 years with normal physical development. (3) Intervention period of at least six weeks.

#### 2.2.2 Exclusion criteria

(1) Literature not meeting inclusion criteria (2) Children with cognitive and behavioral disorders (3) Experimental and control groups with missing basic information (4) Duplicated literature (5) Lack of full-text literature (6) TGMD-2 not used as a measurement tool.

#### 2.2.3 Interventions

The experimental group was supervised by different types of physical activity programs. The duration of the intervention ranged from six weeks to two years. The control group participated in physical activity as usual, but the frequency and intensity of the exercises were unlimited. The experimental and control subjects were similar in terms of age, sex ratio, and level of physical activity before the intervention.

#### 2.2.4 Outcome indicators

(1) running ability, using running as a test method; (2) jumping ability, using standing long jump and one-legged jump as test methods; (3) object control ability, using TGMD-2 as a test method, mainly including kicking, catching, striking a stationary ball, overhand throwing, and underhand throwing (because most studies in the retrieved literature used TGMD as the measure, object control under TGMD was selected as the measure in this study); and (4) stability, measured by walking on a balance beam.

### 2.3 Quality assessment and data extraction

The article’s quality assessment and data extraction were mainly carried out by two researchers, with the third researcher participating in the quality assessment and data extraction in case of disagreement. After extraction, the difference between the mean and standard deviation of the experimental and control groups before and after the intervention was calculated. The mean was calculated by subtracting the pre-test from the post-test, and the standard deviation was calculated using the formula “SD difference^2^ = SD baseline^2^ + SD final^2^–2*R*SD baseline * SD final, R = 0.5” (35).

#### 2.3.1 Quality evaluation

**Figure 1:**
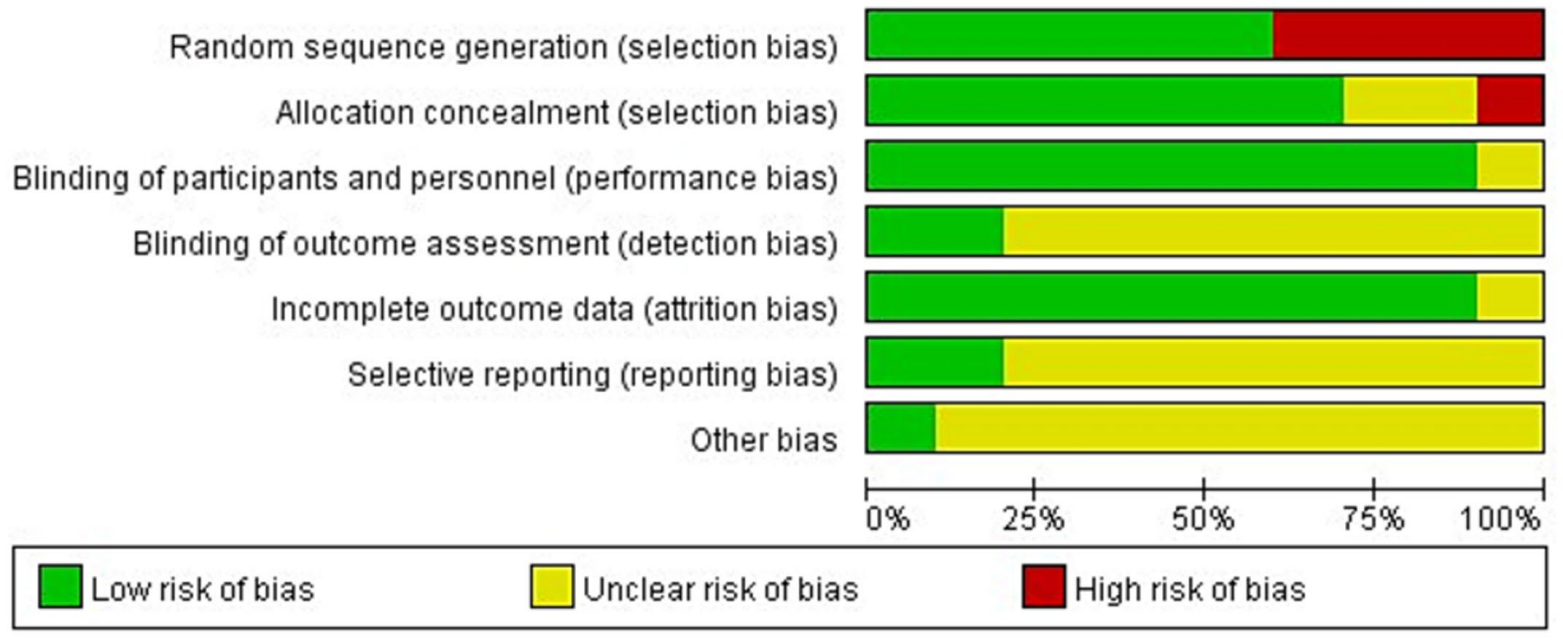
Risk of bias summary for all included studies.

**Figure 2:**
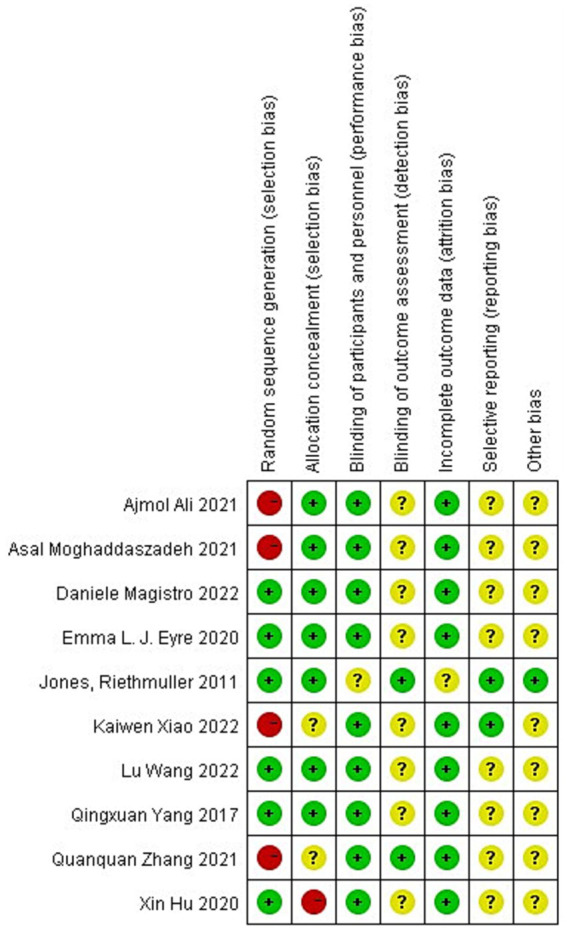
Risk of bias graph of the included studies.

The included literature was scored using the criteria of the Cochrane Handbook of Systematic Evaluation (36) risk of bias assessment scale, which were (1) random sequence generation; (2) allocation concealment; (3) blinding of participants and personnel; (4) blinding of outcome assessment; (5) incompleteness of outcome data;(6) selective reporting; (7) other bias. Each criterion has three options: low bias, high bias, and unclear. When the number of low biases in the literature is ≥4, it is assigned to category A; when the number of low biases in the literature is ≥2 and < 4, it is assigned to category B; and when the number of low biases in the literature is <2, it is assigned to category C. In total, five articles were assigned to Category A (22, 26, 28, 29, 31), and five articles were assigned to Category B (23–25, 27, 30), and the overall quality of the literature was high (see Figures 1, 2).

#### 2.3.2 Statistical analysis

Meta-analysis was conducted using Review Manager 5.4 software (37). The chi-square test was used to determine the heterogeneity between studies, supplemented by I^2^ for quantitative evaluation. An I^2^ value greater than 50% indicated heterogeneity, prompting the use of a random-effects model, whereas an I^2^ value less than 50% signified homogeneity, leading to the selection of a fixed-effects model. Effect sizes were categorized based on the absolute value of the standardized mean difference (SMD): 0–0.4 indicated a small effect, 0.4–0.8 a medium effect, and values greater than 0.8 a large effect (38).

## 3 Results

### 3.1 Results of the literature search

**Figure 3:**
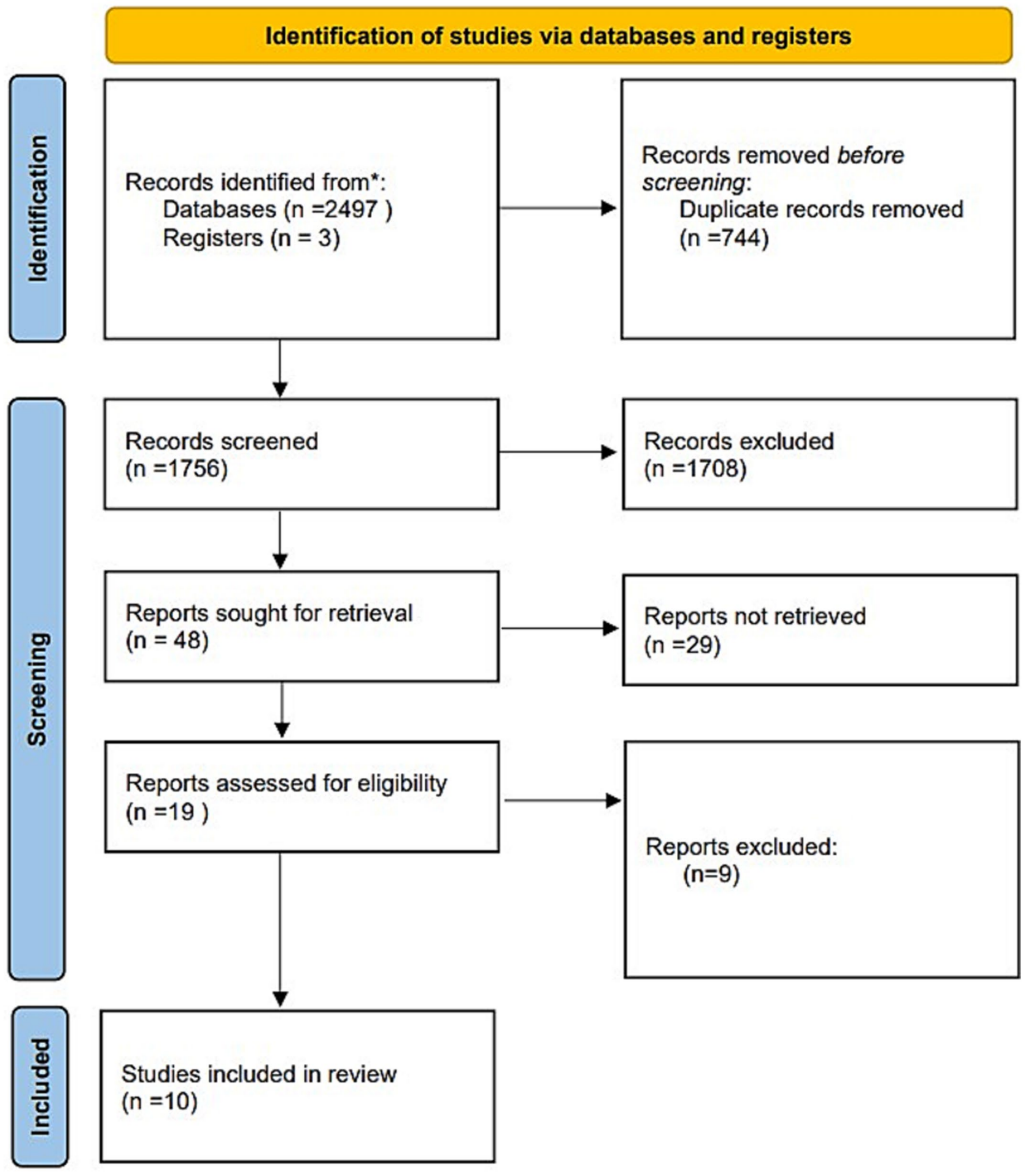
Flow diagram of the selected studies.

Through the search of Chinese and English literature, a total of 2,500 references were retrieved; 744 references were excluded as duplicates, 1,708 references that did not meet the inclusion criteria were excluded after reading the title and abstract, 29 references were excluded after reading the full text, and 10 references were finally excluded, of which a total of 6 references were in English (22–25, 28, 29, 31) and a total of 4 references were in Chinese (26, 27, 30, 31) (see Figure 3).

### 3.2 General information about the included studies

A total of 1,121 children aged 3–7 years were included: 580 children in the experimental group and 541 children in the control group. The interventions were different types of physical activity programs: two differentiated between genders (23, 27), two (25, 27) illustrated the intensity of the interventions, two interventions were conducted in different age groups (24, 26), and four studies had a follow-up survey after the intervention (22, 23, 28, 31) (see Tables 1, 2).

**Table 1:** Status of subjects included in the literature.

Number	Author	Year	Experimental subjects	
			Age (years)	Experimental group (male: female)/Control group (male: female)
1	Jones et al. (22)	2011	3–5	Experimental group: 52 Control group: 45
2	Yang (26)	2017	3–5	Experimental group: 60 Control group: 60
3	Eyre et al. (28)	2020	5–6	Experimental group: 39 Control group: 46
4	Hu et al. (24)	2020	3–5	Experimental group: 142 Control group: 147
5	Zhang (27)	2021	6–7	Experimental group 86 (male: 44, female: 40) Control group: 84 (male: 44, female: 42)
6	Ali et al. (23)	2021	3–4	Experimental group 46 (male: 24, female: 22) Control group: 20 (male: 13, female:8)
7	Moghaddaszadeh (25)	2021	5–7	Experimental group (LOC):17 Experimental group (OC):21 Control group:14
8	Xiao (30)	2022	3–7	Experimental group:45 Control group: 45
9	Lu (31)	2022	5–6	Experimental group:36 Control group:34
10	Magistro et al. (29)	2022	5–7	Experimental group:36 Control group:46

**Table 2:** Status of interventions included in the literature.

Number	Author	Intervention			
		Training content and ratios	Group: total time/weekly frequency/session duration	Test contents	Training intensity
1	Jones et al. (22)	EG: Jump Start (5 min for mat time, 35 min for equipment, 5 min for cooldown) CG: Normal physical activity programs	EG: 20-week /3times/20 min CG: 20-week	Run/Hop Jump/Catch Kick	Unknown
2	Yang (26)	EG: Core Movement Experience Teaching Activity CG: Traditional sports teaching activities	EG: 12 weeks/2 times/30 ~ 40 min CG: 12 weeks/2 times/30 ~ 40 min	Run/Gallop Skip/Hop Horizontal jump Slide/Dribble Catch/Kick Two-hand strike Overhand throw Underhand throw One hand strike Locomotor skill Object control skill	Unknown
3	Eyre et al. (28)	EG: movement and storytelling intervention; (a) warm up and introduction (6 min); (b) skill stations (3 × 6 min with children rotating stations) or whole group activity (2 × 9 min); and (c) cool down and closure of skill instruction (6 min) CG: the normal Physical Education curriculum	EG: 12-week /35 min CG: 12-week	Run/Jump Stationary dribble Catch/Kick Locomotor skill Object control skill	Unknown
4	Hu et al. (24)	EG: novel rhythmic physical activities (NRPA as an acronym) CG: traditional rhythmic physical activities (TRPA as an acronym)	EG: 1 year /5times/30 min CG: 1 year	Run/ Leap Horizontal jump Hop/Gallop Slide/Dribble Kick/Catch Strike/Throw Underhand rolling Locomotor skill Object control skill	Unknown
5	Zhang (27)	EG1: Moderate to high-intensity physical activity (50%) EG2: Moderate to high-intensity physical activity (60%)	EG1: 14 weeks/2 times/45 min EG2: 14 weeks/2 times/45 min	Run/Gallop Skip/Hop Horizontal jump Slide Two-hand strike One hand strike Dribble/Catch Kick Overhand throw Underhand throw Locomotor skill Object control skill	EG1(intensity accumulation 20 min) EG2 (intensity accumulated 24 min)
6	Ali et al. (23)	EG: Jumping Beans (5 min for mat time, 35 min for equipment, 5 min for cool down) CG: Normal physical activity programs	EG: 10 weeks /1 time/45 min CG: 10 weeks	Locomotor skill Object control skill	Unknown
7	Moghaddaszadeh (25)	EG1: guided active play locomotor EG2: guided active play object control CG: the active play	EG1: seven weeks /4 times/one hour CG: seven weeks/four times/one hour	Locomotor skill Object control skill	Unknown
8	Xiao (30)	EG: Sports Intervention program CG: Normal sports program	EG:10 weeks/2times ≤ 30 min CG:10 weeks	Run/ Hop Horizontal jump Skip/Gallop Slide/Dribble Catch/Kick Two-hand strike Overhand throw Underhand throw Locomotor skill Object control skill	30–40%
9	Lu (31)	EG: Early Childhood Football Program CG: Normal Physical Education Program	EG:12 weeks/3 times/30 min CG:12 weeks	Run horizontal jump	Unknown
10	Magistro et al. (29)	EG: physically active mathematics lessons CG: PE curriculum and mathematics curriculum	EG: Two years CG: Two years/PE curriculum was 1 h per week, and mathematics curriculum was 8 h per week	Locomotion skill object control – ball skills	Unknown

EG is the experimental group; CG is the control group.

## 4 Meta-analysis results

### 4.1 Running ability

**Figure 4:**
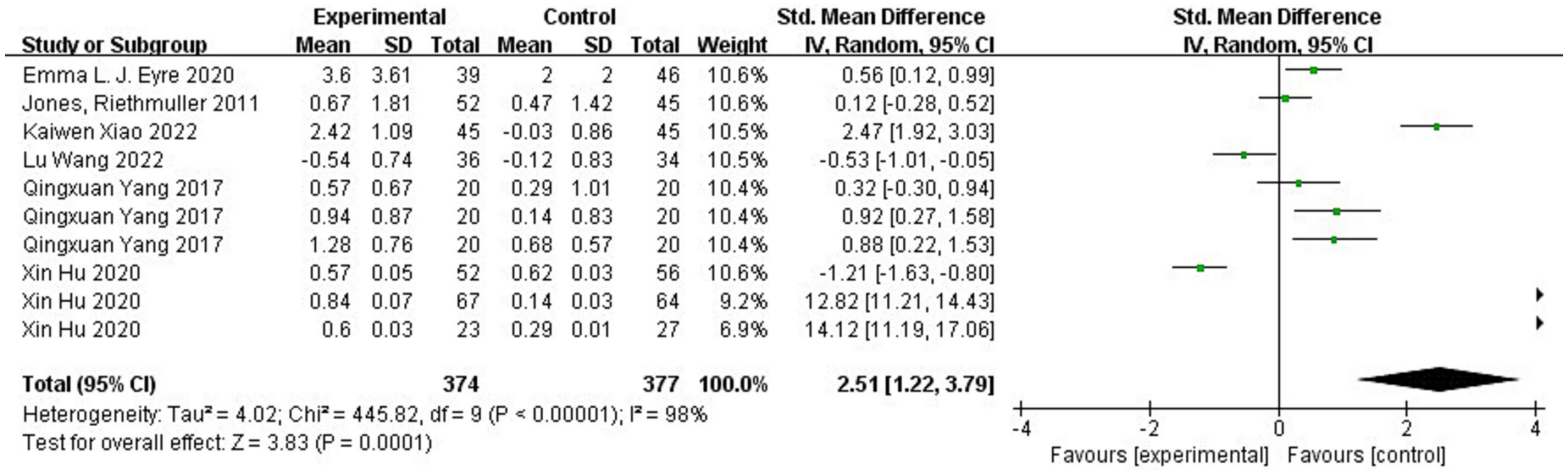
Forest plot of the running ability of experimental and control groups after intervention.

**Figure 5:**
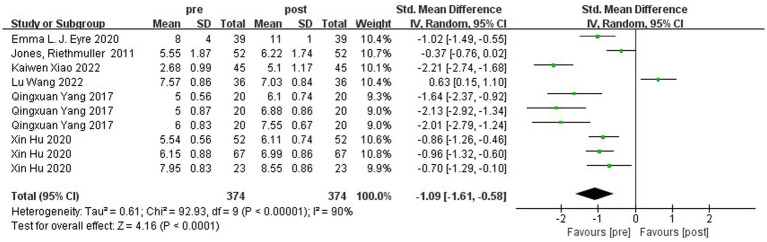
Forest plot of the running ability of the experimental group before and after the intervention.

A total of six pieces of literature have studied running ability in this study. Figure 4 shows the forest plot of running ability between the experimental and control groups after the intervention, χ^2^ = 445.82, df = 9 (*p* < 0.00001), and I^2^ = 98% between the experimental and control groups. It can be assumed that there is heterogeneity between the two groups, and meta-analysis was conducted using the random effects model. The results showed SMD = 2.51, 95% CI [1.22. 3.79], *p* = 0.0001, which is statistically significant when combined, and the diamond is to the right of the null line, indicating that the difference between the groups is statistically significant, suggesting that physical activity improves children’s running ability. Figure 5 shows that χ^2^ = 92.93, df = 9 (*p* < 0.00001), I^2^ = 90%, SMD = −1.09, 95% CI [−1.61, −0.58], *p* < 0.00001, indicating that there is a significant change in running ability before and after the intervention in the experimental group.

### 4.2 Jumping ability

**Figure 6:**
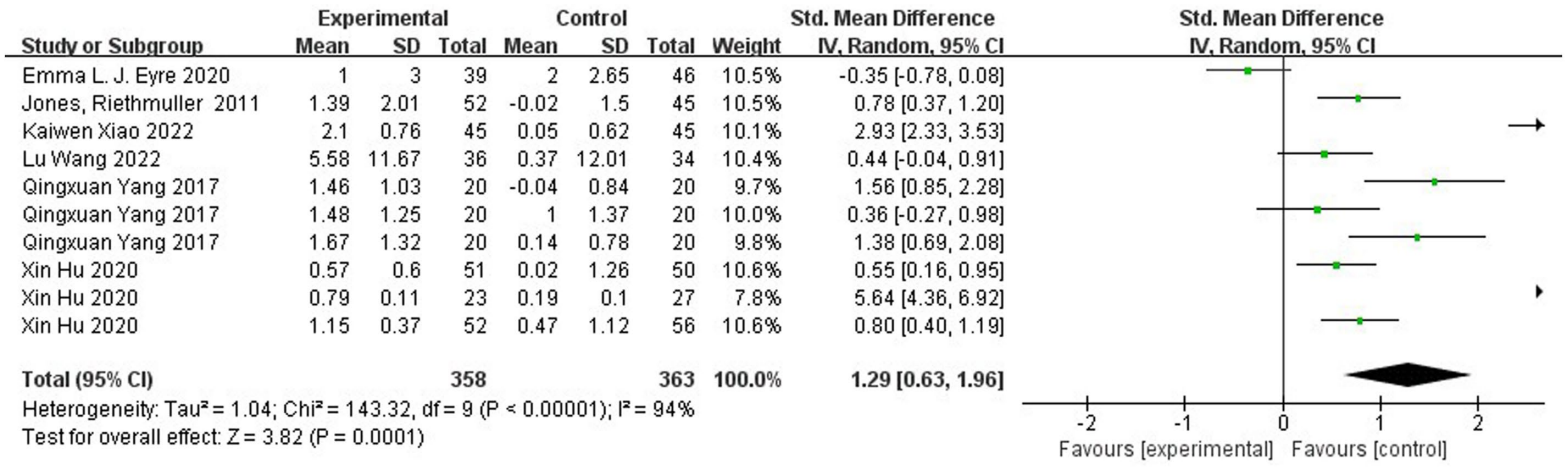
Forest plot comparing horizontal jump of the experimental and control groups after the intervention.

**Figure 7:**
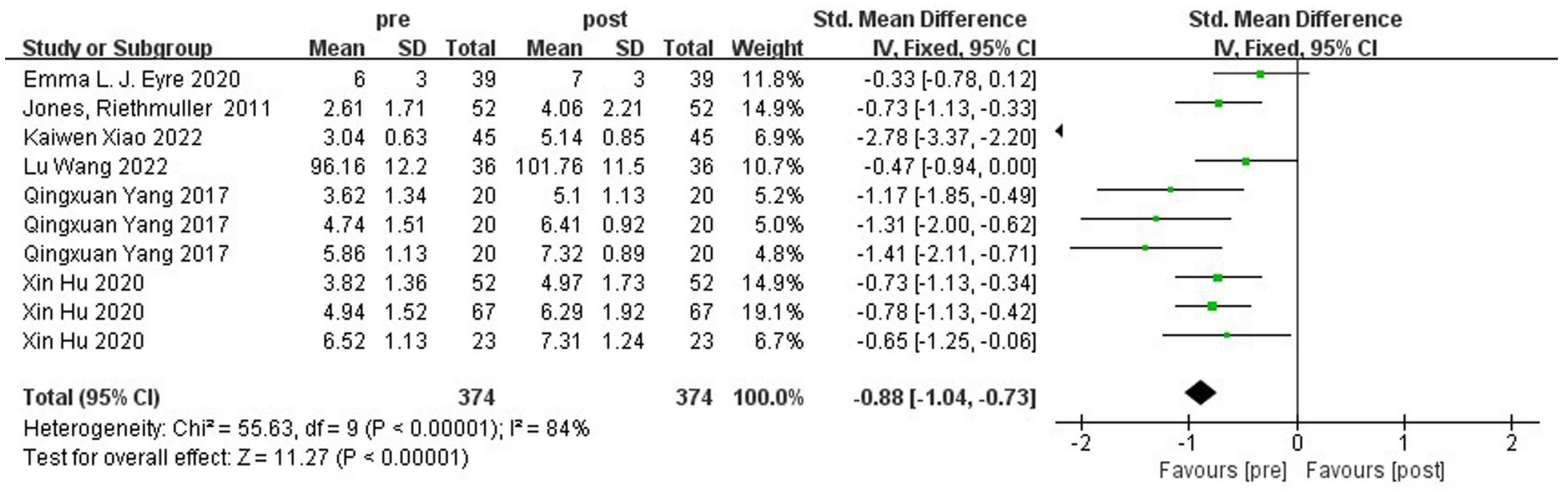
Forest plot comparing the horizontal jump of the experimental group before and after the intervention.

Jumping ability was mainly measured by horizontal jump and hop ability. Figure 6 shows the forest plot of the horizontal jump ability of the experimental group and the control group after the intervention, χ^2^ = 143.32, df = 9 (*p* < 0.00001), I^2^ = 94% between the experimental group and the control group. It can be assumed that there is heterogeneity between the two groups, and meta-analysis was performed using the random effects model. SMD = 1.29, 95% CI [0.63, 1.96], *p* = 0.0001, the diamond is to the right of the null line, with a high degree of effect, indicating that the difference between the groups is statistically significant, suggesting that the different types of physical activity programs have an effect on improving children’s jumping ability. As can be seen in Figure 7, the horizontal jump χ^2^ = 55.63, df = 9 (*p* < 0.00001), I^2^ = 84%, SMD = −0.88, 95% CI [−1.04, −0.73], *p* < 0.00001 in the experimental group, indicating that there was a significant change in the horizontal jump ability of the experimental group before and after the intervention.

**Figure 8:**
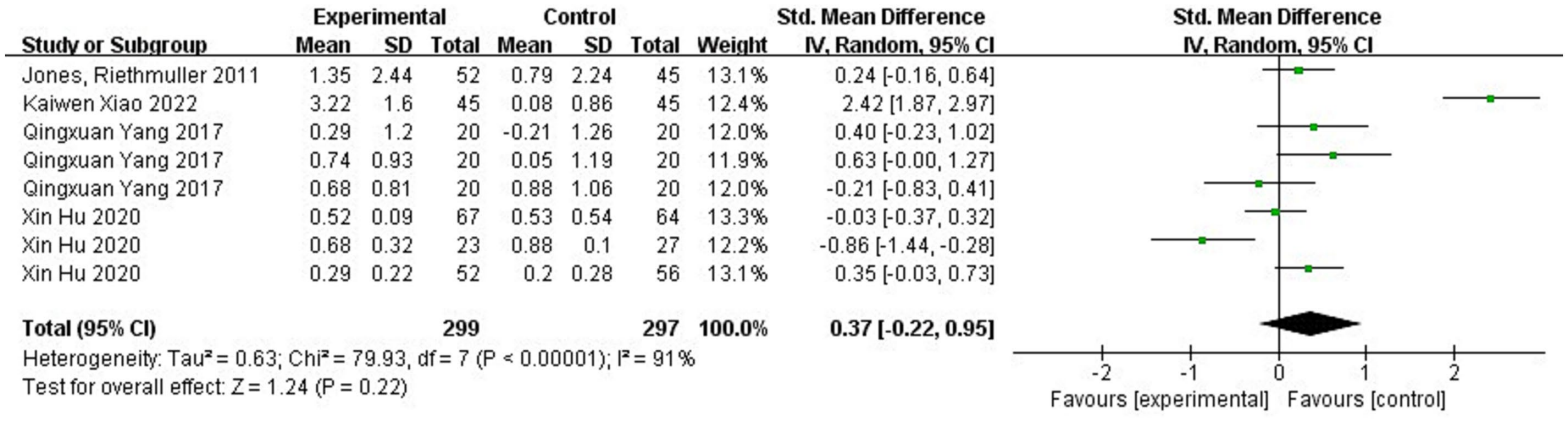
Forest plot comparing the hop ability of the experimental and control groups after the intervention.

**Figure 9:**
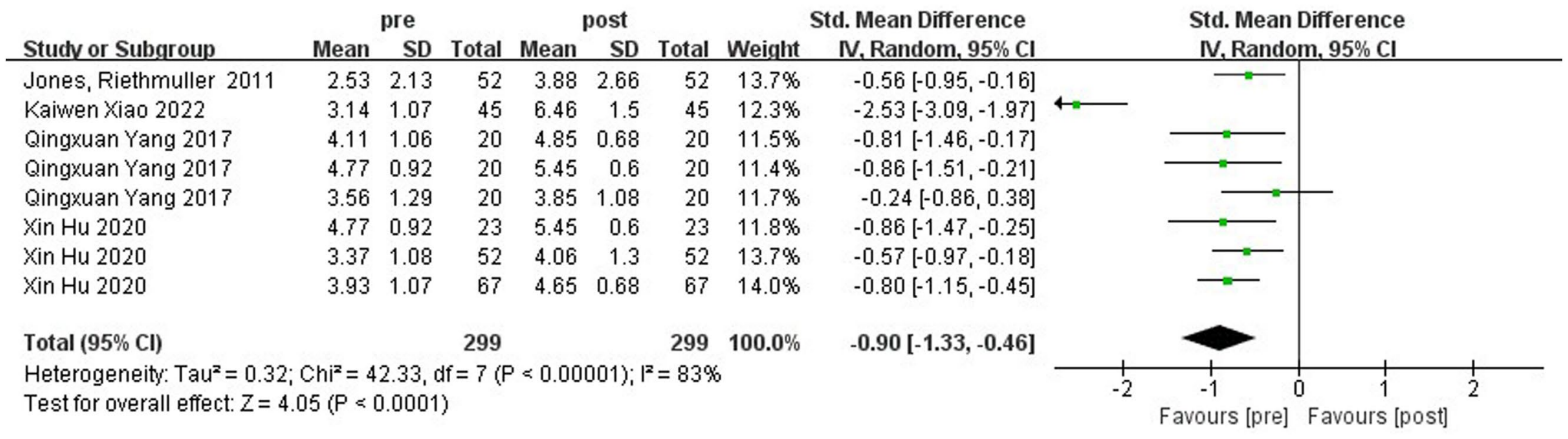
Forest plot comparing the hop ability of the experimental group before and after intervention.

Figure 8 shows the forest plot of hop ability between the experimental and control groups after the intervention. χ^2^ = 79.93, df = 7 (*p* < 0.00001), I^2^ = 91% between the experimental group and the control group, it can be considered that there is heterogeneity between the two groups, and meta-analysis was carried out using the random-effects model. SMD = 0.37, 95% CI [−0.22, 0.95], *p* = 0.22, and the rhombus intersects the null line, indicating that the difference between the groups was not statistically significant and that different kinds of physical activity programs were not able to promote children’s one-legged jumping ability. As can be seen from Figure 9, the χ^2^ = 42.33, df = 7 (*p* < 0.00001), I^2^ = 83%, SMD = −0.90, 95% CI [−1.33, −0.46], *p* < 0.0001, indicating that there was a significant change in hop ability before and after the intervention in the experimental group.

### 4.3 Locomotion skill

**Figure 10:**
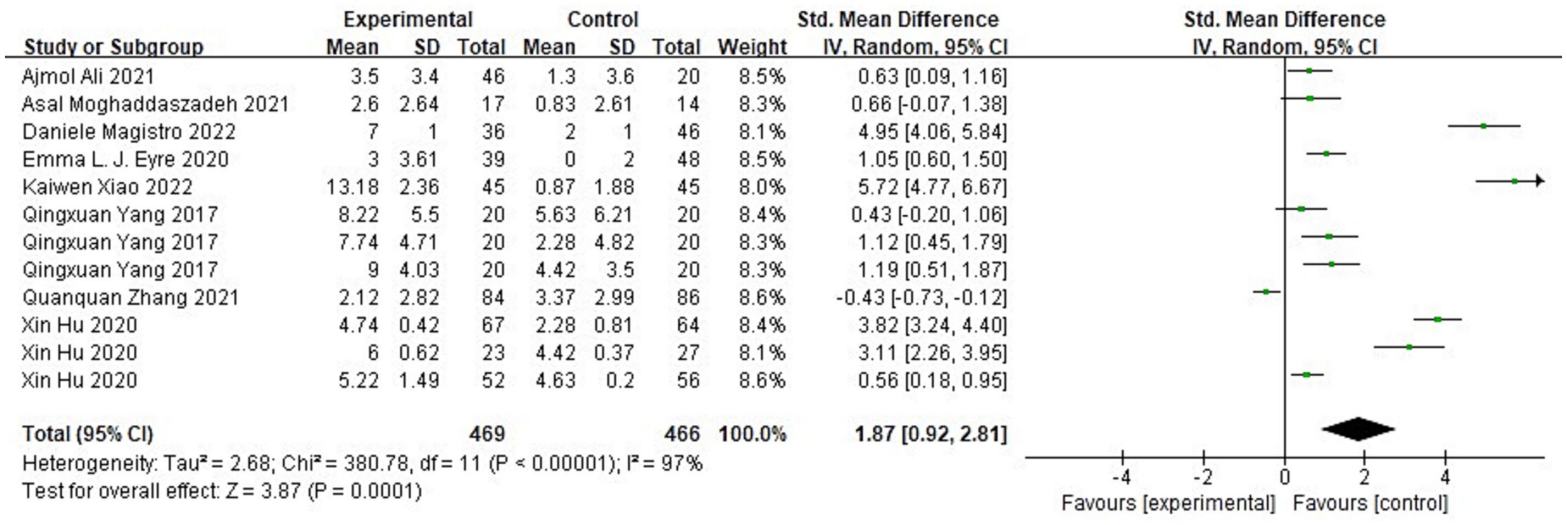
Forest plot of locomotion skill of the experimental and control groups after the intervention.

**Figure 11:**
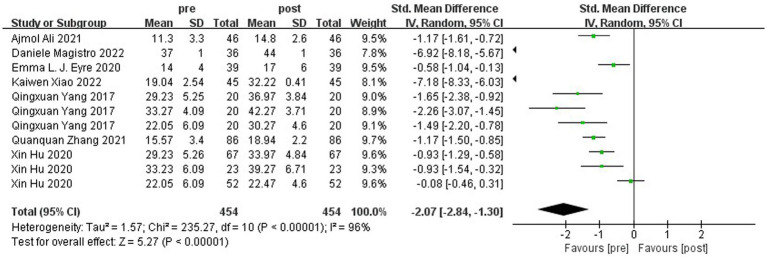
Forest plot of locomotion skill of the experimental group before and after the intervention.

Figure 10 shows the forest plot of locomotion skills between the experimental group and the control group after the intervention. χ^2^ = 380.78, df = 11 (*p* < 0.00001), I^2^ = 97% between the experimental group and the control group, it can be considered that there is heterogeneity between the two groups, and meta-analysis was carried out using the random effects model. SMD = 1.87, 95% CI [0.92, 2.81], *p* = 0.0001, the diamond is located on the right side of the null line with a high effect, indicating that different types of physical activity programs have a better-facilitating benefit on children’s overall displacement ability. Figure 11 shows that the experimental group χ^2^ = 235.27, df = 10 (*p* < 0.00001), I^2^ = 96%, SMD = −2.07, 95% CI [−2.84, −1.30], *p* = 0.0001, and the rhombus is located on the left side of the null line, suggesting that there is a significant change in overall displacement ability before and after the experimental group.

### 4.4 Object control ability

**Figure 12:**
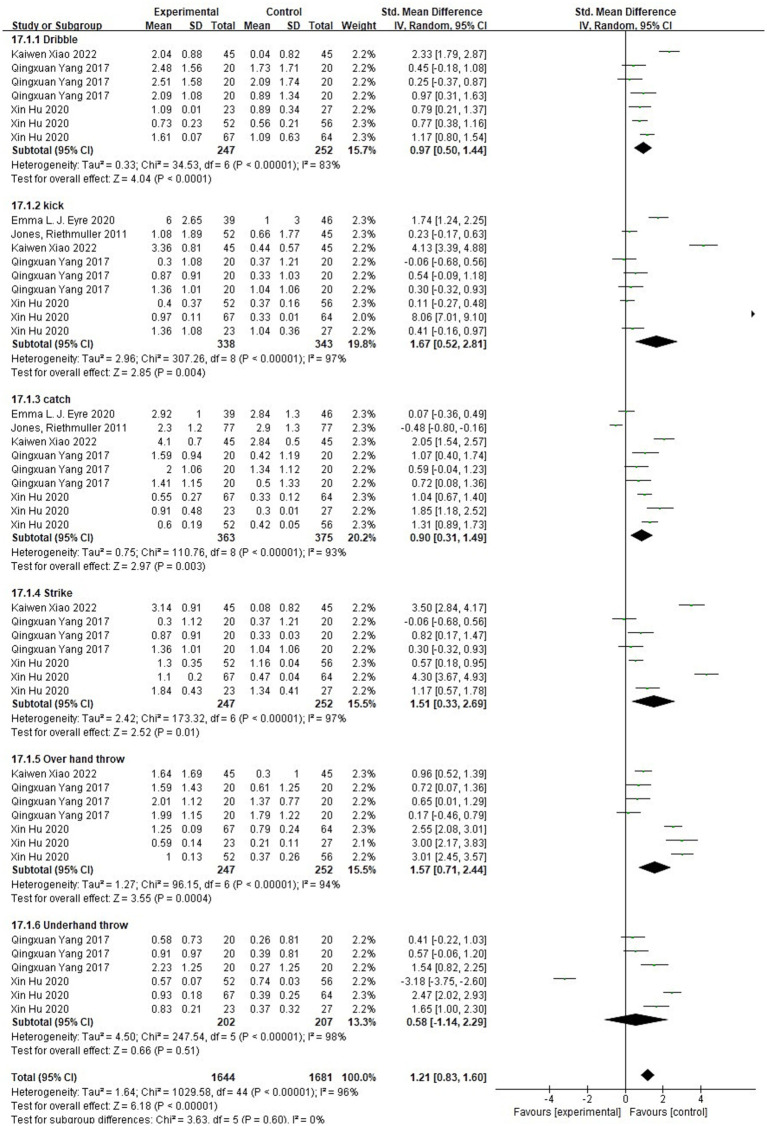
Forest plot comparing object control in the experimental and control groups after the intervention.

As can be seen in Figure 12, the heterogeneity of the effect of the physical activity program on children’s object control ability was χ^2^ = 1029.58, df = 44 (*p* < 0.00001), I^2^ = 96%, and therefore, meta-analysis was conducted using a random effects model with SMD = 1.21, 95% CI [0.83, 1.60], *p* < 0.00001, and SMD > 0.8, which indicated that the physical activity program intervention promoted children’s object control better and there were significant differences between subgroups.

Heterogeneity in dribbling the ball was observed (χ^2^ = 34.53, df = 6, *p* < 0.00001, I^2^ = 83%), indicating substantial heterogeneity. A random-effects model was applied, yielding an SMD of 0.97 (95% CI [0.50, 1.44], *p* < 0.00001). The diamond-shaped squares were positioned to the right of the null line, suggesting that the physical activity program significantly enhanced the promotion of *in-situ* racket skills.

Heterogeneity in kicking the ball was observed (χ^2^ = 307.26, df = 8, *p* < 0.00001, I^2^ = 97%), indicating high heterogeneity. A random-effects model was applied, resulting in an SMD of 1.67 (95% CI [0.52, 2.81], *p* = 0.004). The diamond-shaped squares were positioned to the right of the null line, indicating that the physical activity sessions significantly improved the ability to kick the ball.

Heterogeneity in catching the ball was observed (χ^2^ = 110.76, df = 8, *p* < 0.00001, I^2^ = 93%), indicating substantial heterogeneity. A random-effects model was applied, yielding an SMD of 0.90 (95% CI [0.31, 1.49], *p* = 0.003). The diamond-shaped squares were positioned to the right of the null line, suggesting that the physical activity sessions significantly improved two-handed catching ability.

Heterogeneity in striking a stationary ball was noted (χ^2^ = 173.32, df = 6, *p* < 0.00001, I^2^ = 97%), indicating high heterogeneity. A random-effects model was used, resulting in an SMD of 1.51 (95% CI [0.33, 2.69], *p* = 0.01). The diamond-shaped squares were located to the right of the null line, indicating that physical activity sessions significantly enhanced the ability to strike a stationary ball.

Heterogeneity in overhand throwing was observed (χ^2^ = 96.15, df = 6, *p* < 0.00001, I^2^ = 94%), indicating substantial heterogeneity. A random-effects model was applied, resulting in an SMD of 1.57 (95% CI [0.71, 2.44], *p* = 0.0004). The diamond-shaped squares were positioned to the right of the null line, suggesting that the physical activity sessions significantly improved overhand throwing ability.

Heterogeneity in underhand throwing was observed (χ^2^ = 247.54, df = 5, *p* < 0.00001, I^2^ = 98%), indicating substantial heterogeneity. A random-effects model was applied, resulting in an SMD of 0.58 (95% CI [−1.14, 2.29], *p* < 0.00001). The rhombus-shaped squares intersected the null line, suggesting that the physical activity sessions did not significantly improve underhand throwing ability.

### 4.5 Stability

**Figure 13:**
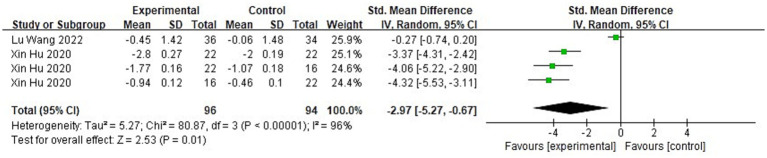
Forest plot comparing object control in the experimental and control groups after the intervention.

**Figure 14:**
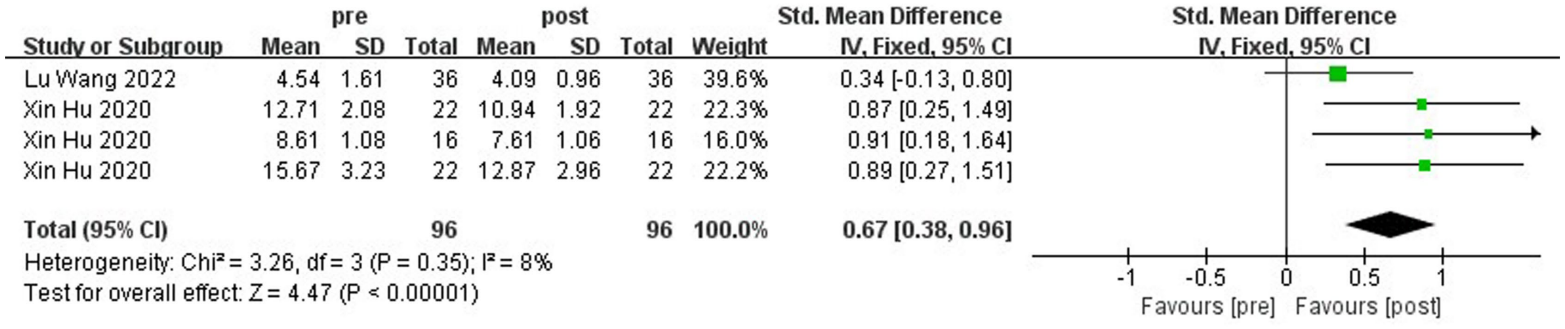
Forest plot of the dynamic balance ability of the experimental group before and after the intervention.

A total of two literature tests on stability ability were included in this study, and the balance beam walking method was used to assess children’s dynamic balance ability. Figure 13 shows the forest plot of the dynamic balance ability between the experimental and control groups after the intervention. χ^2^ = 80.87, df = 3 (*p* < 0.00001), and I^2^ = 96% between the experimental group and the control group, which can be regarded as the existence of heterogeneity between the two groups, and the random effect model was used to Meta-analysis was performed. SMD = −2.97, 95% CI [−5.27, −0.67], *p* = 0.01, the diamond is located on the left side of the null line, which indicates that physical activity has a good benefit in promoting children’s stability ability. As can be seen in Figure 14, χ^2^ = 3.26, df = 3 (*p* = 0.35), I^2^ = 8%, SMD = 0.67, 95% CI [0.38, 0.96], *p* < 0.00001, indicating that the experimental group’s balancing ability was improved after the intervention.

## 5 Sensitivity analysis

Due to the variation in the content and methodology of the literature cited, sensitivity analysis was conducted to assess the effect of this heterogeneity. In the analysis of heterogeneity for each motor ability, the results showed that the I^2^ for running ability, horizontal jump, hop ability, locomotion skill, dribbling, catching, kicking, striking, overhand throwing, and underhand throwing ability remained high after excluding the selected literature one by one, but it did not affect the stability of the experimental results. This means that although there were differences between the different studies, their effect on the overall effect size was not significant, suggesting that the results were relatively robust.

In the heterogeneity analysis of balance ability, after excluding Lu’s study (31), the I^2^ dropped significantly from 96 to 0%, suggesting that this study might be a major source of heterogeneity. To further explore the reasons for heterogeneity differences, we conducted an in-depth analysis from three aspects: the intervention participants, content, and duration. Regarding the intervention participants, Lu’s study (31) involved children aged 5–6, while Hu X’s study included children aged 3–6 (24), dividing them into three age groups for comparison. As for the intervention content, Lu’s study (31) used toddler soccer training, mainly focusing on skills such as dribbling, ball control, trapping, and passing to develop children’s FMS. The course was more geared towards reaction and speed, with balance ability being applied but not as a core focus of the curriculum. As a result, the improvement in balance ability may not have been significant. In contrast, Hu et al.’s study (24) used a novel rhythmic exercise as the intervention method. This new rhythmic exercise was built upon traditional rhythmic movements (such as flexing, vibrating, twisting, and circling, which aid in the development of balance ability) and incorporated exercises for locomotor and object control abilities. This more systematic and comprehensive intervention method likely placed greater emphasis on improving balance ability, which might explain the more significant effects observed in this study. In terms of intervention duration, the duration of Hu et al.’s study (24) was longer than that of Lu’s study (31), giving children more time to participate and practice. A longer intervention period generally provides more opportunities for children to reinforce newly acquired skills. Particularly for balance, which requires repetitive practice and fine motor control, increased time might have had a substantial impact.

## 6 Subgroup analyses

As less of the included literature measured balance ability, which was not suitable for subgroup analysis, this study analyzed subgroups in terms of weekly intervention duration and intervention period as well as different age groups. When comparing the performance of children in the two subgroups with weekly intervention durations of 90 min and below and 90 min and above in various basic motor skill tests, the results showed that SMD90min above > SMD90min below in running ability, horizontal jump, and catching ability, suggesting that the effect of the weekly intervention durations of 90 min and above for running ability, horizontal jump, and catching ability is better than that of below 90 min. In kicking ability, although SMD above 90 min = 2.81 is a large effect size, it is not a significant difference, which may be related to the training method or individual differences, and more data or further research is needed to confirm this (see Table 3).

**Table 3:** Comparison of the effects of different weekly intervention durations.

Test indicators	Subgroups	Number of parts	*Z*	*P*	SMD 95%CI
Run	More than 90 min	2	2.90	0.004	6[1.94,10.06]
	Less than 90 min	4	2.49	0.010	0.87[0.18, 1.56]
Horizontal jump	More than 90 min	2	2.97	0.003	2.25[0.77, 3.74]
	Less than 90 min	4	2.33	0.020	1.1[0.17,2.02]
Kick	More than 90 min	1	1.61	0.110	2.81[−0.61,6.24]
	Less than 90 min	4	2.07	0.040	1.13[0.06, 2.21]
Catch	More than 90 min	1	6.5	<0.010	1.32[0.92, 1.72]
	Less than 90 min	4	1.62	0.110	0.66[−0.14, 1.45]

P retains three decimal places.

This study divided the intervention cycle into less than 12 weeks, 12 weeks, and more than 12 weeks. The results showed that the intervention effect of less than 12 weeks was better than 12 weeks and more for running ability, horizontal jump, kicking ability, and catching ability; kicking ability was not significant at 12 weeks. There were no between-group differences in catching ability beyond 12 weeks. This study suggests that further observations are needed to draw accurate conclusions about the mastery of object control skills (see Table 4).

**Table 4:** Comparison of effects of different intervention cycles.

Test indicators	Subgroups	Number of parts	*Z*	*P*	SMD 95%CI
Run	More than 12 weeks	1	1.95	0.050	3.74 [−0.01, 7.49]
	12 weeks	3	3.08	<0.010	2.22 [0.81, 3.64]
	Less than 12 weeks	1	8.75	<0.010	2.47[1.92, 3.03]
Horizontal jump	More than 12 weeks	2	3.32	<0.010	2.32 [0.95, 3.69]
	12 weeks	2	2.94	<0.010	0.90 [0.30, 1.50]
	Less than 12 weeks	1	9.54	<0.010	2.93[2.33, 3.53]
Kick	More than 12 weeks	2	1.98	0.050	2.13 [0.02, 4.24]
	12 weeks	2	1.52	0.130	0.65 [−0.19, 1.48]
	Less than 12 weeks	1	10.9	<0.010	4.13 [3.39,4.88]
Catch	More than 12 weeks	2	1.74	0.080	0.91 [−0.11, 1.93]
	12 weeks	2	2.44	0.010	0.56 [0.11, 1.02]
	Less than 12 weeks	1	7.82	<0.010	2.05 [1.54, 2.57]

*P retains three decimal places.*

This study compares the fundamental movement skills of 3–5 and 5–7 year-olds separately. 3-5-year-olds showed a medium and significant difference in effect sizes for running ability, horizontal jump, and catching, while 5-7-year-olds did not show a significant difference for running ability, horizontal jump, and catching. 3-5-year-olds showed a statistically significant difference in kicking ability with a medium effect size, and 5-7-year-olds were more prominent in this indicator with a significant difference and a large effect size, indicating a significant increase in kicking ability for this age group. Children showed more prominent performance on this indicator with a significant difference and large effect size, indicating that children in this age group showed significant improvement in kicking ability (see Table 5).

**Table 5:** Comparison of intervention effects by age.

Test indicators	Subgroups	Number of parts	*Z*	*P*	SMD 95%CI
Run	3–5 years	3	2.08	0.040	0.50 [0.03, 0.96]
	5–7 years	4	1.37	0.170	0.55 [−0.24, 1.33]
Horizontal jump	3–5 years	3	4.72	<0.010	0.47 [0.28, 0.67]
	5–7 years	3	1.02	0.310	0.47 [−0.43, 1.37]
Kick	3–5 years	3	2.18	0.030	0.32 [0.03, 0.61]
	5–7 years	3	2.26	0.020	0.87 [0.11, 1.62]
Catch	3–5 years	3	2.86	0.004	0.51 [0.16, 0.86]
	5–7 years	3	1.62	0.110	0.41 [−0.09, 0.91]

*P retains three decimal places.*

## 7 Discussion

Some studies have confirmed that physical activity programs can improve FMS levels. Ali et al. (23) found significant improvements in children’s FMS levels through a ten-week physical activity program intervention. Jones et al. (22) made a similar observation in 2011 through a twenty-week instrumental intervention. However, there was a bias in children’s jumping ability, which could be attributed to the different focuses of different physical activity programs and the different physical fitness of the children involved in the experiment, resulting in a bias in results. This may be due to the program’s different focuses and the physical qualities of the children involved in the experiment. Magistro et al. (29) proposed in their study “classroom-based physical activity,” a new type of physical activity program that combines math lessons with physical activity by starting each lesson with a 10-min running walk and other physical activities. At the beginning of each lesson, the teacher warms up with 10 min of physical activity such as running and walking. Then, in the explanation section, the teacher explains the mathematical concepts for 15 min, demonstrates the physical activity learning task, guides the children to apply the mathematical knowledge explained in the explanation section through the physical activity learning task, and then concludes the lesson. Through the integrated teaching of education and physical activity, children’s potential to improve their knowledge and skills is fully realized.

Different types of physical activity programs have a positive effect on the improvement of children’s displacement ability, which mainly consists of running ability and jumping ability, and when analyzing the effect of different physical activity programs on running ability, children’s running ability was significantly improved after the intervention, as evidenced by relevant studies (22, 26, 28, 30, 31), mainly because the running ability is one of the earliest abilities to emerge in children, however, Hu et al. (24) found in his study that after a period of intervention, the running ability of the 3-year-old experimental group was lower than the running ability of the control group, probably because some of the children at that time are still in the early stages of development, their coordination and muscle control may not have reached the level of their peers, and some of the children may have already developed better coordination and muscle control, leading to the differences in results.

When analyzing the impact of physical activity on horizontal jump, it was observed that children’s horizontal jump ability significantly improved after the intervention. However, Eyre et al.’s study (28) noted that children in the experimental group exhibited lower horizontal jump ability than those in the control group. This disparity may stem from demographic differences between the two groups. Notably, in Eyre et al.’s study (28), the experimental group was comprised predominantly of South Asian children, while the control group consisted mostly of white children. Such population variances might have influenced the outcomes due to diverse cultural and genetic backgrounds, thereby impacting the children’s athletic performance. Previous research (22, 30) has demonstrated a significant enhancement in hop ability following a training period. However, Hu et al. (24) revealed no significant discrepancy in hop ability between the experimental and control groups at the ages of 3 and 5 years post-intervention. This outcome could be attributed to the inherent demands of hop exercises, which necessitate greater muscle strength, multi-limb coordination, and dynamic balance compared to vertical jumps for proficient execution. Moreover, mastering jumping skills typically requires considerable time and practice. Furthermore, jumping entails a multifaceted task requiring extensive skill development, demanding children to possess adequate strength and coordination between their arms and legs (39).

The physical activity program successfully enhanced children’s object control skills, yet no significant improvement was observed in hand-throwing. This aligns with the findings of Hu et al. (24), where the hand-throwing ability of the 4-year-old group showed no notable change over time, possibly due to irregular movements and a lack of coordination. Regarding catching the ball, Jones et al. (22) discovered that children’s proficiency in two-handed catching did not significantly improve after a specific intervention period. This might be attributed to the study’s emphasis on enhancing children’s displacement ability rather than giving sufficient attention to the development of object control skills, leading to a lack of significant progress in this aspect of performance.

As this study has limited literature regarding balance skills, the analysis of such skills may be less precise, and further validation is necessary in the future.

Most of the studies had no more than 12 weeks of intervention. The duration of the intervention tended to be no more than 90 min per week, so this study argues that a longer duration of intervention does not necessarily lead to better exercise performance, which is in line with the conclusions reached by Li et al. (40). Additionally, a meta-analysis on the effects of physical activity programs on gross motor skill development in preschool children reached a similar conclusion (41). Except for interventions lasting longer than 12 weeks, other interventions with varying durations, frequencies, and cycles significantly promoted the development of gross motor skills in young children. This finding suggests that high-frequency and long-duration interventions may reduce children’s adherence and enthusiasm for physical activities. Furthermore, prolonged interventions might be subject to a “ceiling effect,” where further improvements become minimal once a certain developmental threshold has been reached (6).

In addition to intervention duration, exercise intensity is an important factor influencing the effectiveness of physical activity. However, in the literature surveyed, there are few clear ranges of exercise intensity for children aged 3–7 years, which may be due to the greater challenge of monitoring children’s physical activity. When providing physical activity recommendations, the World Health Organization (WHO) has specifically emphasized that a differentiated approach to instruction should be taken for children aged 3–5 years versus 5–6 years (42), and in analyzing the findings of this study, it was found that the level of basic motor skills of children aged 3–5 years was higher than that of children aged 5–7 years, especially in terms of running ability, horizontal jump, and catching ability, and that the kicking ability of 5-7-year-olds performed better, which is contrary to the results obtained by Gao and Wang (43), probably because at the age of 3–5 years, children are usually in the early stages of growth and development, their bodies may be better suited for fast running, jumping and catching the ball, and their muscle control and coordination may also be relatively good at this age. At the same time, their cognitive abilities are developing rapidly, which makes it easier for them to understand and execute simple technical movements. However, by the age of 5–7 years, their cognitive abilities may be developing further, allowing them to better understand and perform more complex skills, and this difference may also be related to their experience of physical activity: 3–5-year-olds are likely to be more involved in basic physical activity, whereas, by the age of 5–7 years, they may be exposed to more types of sport, like ball games, which require more complex skills, and therefore increase competence in these areas. Thus, kicking a ball may require more skill and coordination relative to running, horizontal jumping, and catching, which makes children aged 5–7 perform better in this area. More literature is needed to confirm this, as the methodological approach varies from experiment to experiment and the physical fitness of the children participating.

Based on the findings of this study, future research should prioritize school and community-based efforts. Schools are particularly well-suited for fostering PA and developing FMS, as children spend approximately 50% of their time in school. Building a strong foundation for FMS during childhood is essential (21), as this is a period when children acquire skills more quickly and easily, without the self-consciousness or shame associated with imperfect motor performance. A strong foundation not only breaks down a “proficiency barrier” (44) that exists prior to the different skill acquisition stages of children between the ages of 3 and 7 and 7 and 12 but also allows for the transition of children’s motor skill levels to a more specialized stage in an organized PA program. Schools need to ensure that they provide rich and varied physical activity content that includes a variety of movements such as running, jumping, throwing, and catching and that the activities are fun and interactive to cater to different children’s interests and abilities. Secondly, physical education teachers should receive professional training to effectively instruct and motivate children to promote the development of their motor skills. To ensure effective implementation of the curriculum, schools and communities should also work closely with parents to develop and implement family activity programs to further promote children’s motor development in the family environment. Schools and communities are required to provide children with continuous assessment and monitoring in light of their physical and mental development to provide more useful values for the development of fundamental movement skills by establishing the optimal time, frequency, and period of intervention.

## 8 Limitations and shortcomings

The limited number of literature included in this study and the small number of balance tests may have resulted in an insufficient sample size, limiting to some extent the overall understanding of children’s fundamental movement skills, which could lead to the occurrence of a chance error. Future studies should expand the literature search to include more databases and keywords to increase the number of literature included. The absence of some of the key rubrics in the text had an impact on the credibility of the results, so future articles should be selected to ensure that all important rubrics are included and reported. Meanwhile, sensitivity analyses should be used in literature screening and data extraction to assess the possible impact on the results of studies with missing key rubrics. This further increases the possibility of analytical error due to the differences in experimental populations, interventions, study designs, and outcome assessments across studies. Therefore, multi-group subgroup analyses or sensitivity analyses are conducted to explore the impact of different study characteristics on the results. In terms of literature quality assessment, the assessment methods used may lack sufficient objectivity and systematicity, which may also affect the judgment of literature quality, and multiple risk assessment tools may be used in the future to improve the objectivity and consistency of the assessment. In addition, subgroup analyses of gender differences were not conducted in the study, and in future studies, gender-specific data should be collected and reported, and gender subgroup analyses should be conducted to explore the effects of gender on children’s fundamental movement skills.

## 9 Conclusion

In summary, different types of physical activity programs had a positive impact on promoting children’s FMS in seven main areas: running ability, standing long jump, tapping a ball in place, kicking a ball, catching a ball with both hands, hitting a stationary ball, and overhand throwing, with no significant improvement in one-legged jumping and underhand throwing, and with fewer tests of balance in the included literature, the results obtained need to be further validated. Meanwhile, the subgroup analysis revealed that interventions exceeding 90 min per week significantly positively impacted children’s running ability, horizontal jumping, and catching skills. Additionally, interventions lasting less than 12 weeks showed greater effectiveness than those of 12 weeks or longer in improving running, horizontal jump, kicking, and catching abilities. Children aged 3–5 years performed better in running, horizontal jumping, and catching, while those aged 5–7 years showed better improvements in kicking ability. This suggests that future research should focus more on the duration of interventions and the careful planning of intervention periods to ensure that children’s motor skills are maximized. It also highlights the importance of selecting appropriate physical activity programs tailored to the growth and developmental characteristics of different age groups for the comprehensive development of children. Furthermore, as a critical factor, the intensity of interventions should be explored in greater depth in future studies.

## Data Availability

The original contributions presented in the study are included in the article/Supplementary material, further inquiries can be directed to the corresponding author.

## References

[1] Clark JE. Motor Development In: Ramachandran VS, editor. Encyclopedia of human behavior. New York: Academic Press (1994). 250.

[2] Logan SW Ross SM Chee K Stodden DF Robinson LE. Fundamental motor skills: a systematic review of terminology. J Sports Sci. (2018) 36:781–96. doi: 10.1080/02640414.2017.1340660, PMID: 28636423

[3] Barnett LM Lai SK Veldman SLC Hardy LL Cliff DP Morgan PJ . Correlates of gross motor competence in children and adolescents: a systematic review and meta-analysis. Sports Med. (2016) 46:1663–88. doi: 10.1007/s40279-016-0495-z, PMID: 26894274 PMC5055571

[4] Gallahue David L. “Understanding motor development: Infants, children, adolescents, adults, 7th ed.” New York, NY: McGraw - Hill. (2012).

[5] Burns RD Bai Y Byun W Colotti TE Pfledderer CD Kwon S . Bidirectional relationships of physical activity and gross motor skills before and after summer break: application of a cross-lagged panel model. Sport Health Sci. (2022) 11:244–51. doi: 10.1016/j.jshs.2020.07.001, PMID: 32652233 PMC9068551

[6] Cattuzzo MT dos Santos HR Ré AHN de Oliveira IS Melo BM deSousa MM . Motor competence and health related physical fitness in youth: a systematic review. J Sci Med Sport. (2016) 19:123–9. doi: 10.1016/j.jsams.2014.12.004, PMID: 25554655

[7] Robinson LE Stodden DF Barnett LM Lopes VP Logan SW Rodrigues LP . Motor competence and its effect on positive development a trajectories of health. Sports Med. (2015) 45:1273–84. doi: 10.1007/s40279-015-0351-6, PMID: 26201678

[8] Caspersen CJ Christenson PGM. Physical activity, exercise, and physical fitness: definitions and distinctions for health-related research. Public Health Rep. (1974), 1985) 100:126–31. doi: 10.2307/20056429, PMID: 3920711 PMC1424733

[9] Carson V Lee EY Hewitt L Jennings C Hunter S Kuzik N . Systematic review of the relationships between physical activity and health indicators in the early years (0-4 years). BMC Public Health. (2017) 17:854. doi: 10.1186/s12889-017-4860-0, PMID: 29219090 PMC5753397

[10] Guthold R Stevens GA Riley LM Bull FC. Global trends in insufficient physical activity among adolescents: a pooled analysis of 298 population-based surveys with 1·6 million participants. Lancet Child Adoles Health. (2020) 4:23–35. doi: 10.1016/S2352-4642(19)30323-2, PMID: 31761562 PMC6919336

[11] Paterson DC Ramage K Moore SA Riazi N Tremblay MS Faulkner G. Exploring the impact of COVID-19 on the movement behaviors of children and youth: a scoping review of evidence after the first year. Sport Health Sci. (2021) 10:675–89. doi: 10.1016/j.jshs.2021.07.001, PMID: 34237456 PMC8687706

[12] Dunton GF Do B Wang SD. Early effects of the COVID-19 pandemic on physical activity and sedentary behavior in children living in the U.S. BMC Public Health. (2020) 20:1351. doi: 10.1186/s12889-020-09429-3, PMID: 32887592 PMC7472405

[13] Morrow Jr JR Jackson AW Payne VG. Physical activity promotion and school physical education In: President’s Council on Physical Fitness and Sports Research Digest (1999)

[14] Jones D Innerd A Giles EL Azevedo LB. Association between fundamental motor skills and physical activity in the early years: a systematic review and meta-analysis. J Sport Health Sci. (2020) 9:542–52. doi: 10.1016/j.jshs.2020.03.001, PMID: 33308805 PMC7749255

[15] Stodden DF Goodway JD Langendorfer SJ Roberton MA Rudisill ME Garcia C . A developmental perspective on the role of motor skill competence in physical activity: An emergent relationship. Quest. (2008) 60:290–306. doi: 10.1080/00336297.2008.10483582

[16] Holfelder B Schott N. Relationship of fundamental movement skills and physical activity in children and adolescents: a systematic review. Psychol Sport Exerc. (2014) 15:382–91. doi: 10.1016/j.psychsport.2014.03.005

[17] Figueroa R An R. Motor skill competence and physical activity in preschoolers: a review. Matern Child Health J. (2017) 21:136–46. doi: 10.1007/s10995-016-2102-1, PMID: 27417826

[18] Barnett L Webster E Hulteen R De Meester AD Valentini N Lenoir M . Correction to: through the looking glass: A systematic review of longitudinal evidence, providing new insight for motor competence and Health. Sports Med (Auckland, N.Z.). (2021) 52:921. doi: 10.1007/s40279-021-01563-1 PMC9172687 34524656

[19] Barnett LM Salmon J Hesketh KD. More active pre-school children have better motor competence at school starting age: an observational cohort study. BMC Public Health. (2016) 16:1068. doi: 10.1186/s12889-016-3742-1, PMID: 27724941 PMC5057262

[20] Wick K Leeger-Aschmann CS Monn ND Radtke T Ott LV Rebholz CE . Interventions to promote fundamental movement skills in childcare and kindergarten: a systematic review and Meta-analysis. Sports Med. (2017) 47:2045–68. doi: 10.1007/s40279-017-0723-1, PMID: 28386652 PMC5603621

[21] Ma R Song H. Effects of fundamental movement skill development on physical activity and health of children. Sports Science (in Chinese). (2017) 37:54–61+97. doi: 10.16469/j.css.201704007

[22] Jones RA Riethmuller A Hesketh K . Promoting movement skill development and physical activity in early childhood settings: a pilot randomised controlled trial. Obes Res Clin Pract. (2010) 4:S17–7. doi: 10.1016/j.orcp.2010.09.034, PMID: 22109783

[23] Ali A Mclachlan C Mugridge O . The effect of a 10-week physical activity Programme on fundamental movement skills in 3–4-year-old children within early childhood education Centres. Children. (2021) 8:440. doi: 10.3390/children8060440, PMID: 34073725 PMC8225089

[24] Hu X Jiang GP Ji ZQ Pang B Liu J. Effect of novel rhythmic physical activities on fundamental movement skills in 3- to 5-year-old children. Biomed Res Int. (2020) 2020:1–10. doi: 10.1155/2020/8861379, PMID: 33426079 PMC7781705

[25] Moghaddaszadeh A Belcastro AN. Guided active play promotes physical activity and improves fundamental motor skills for school-aged children. J Sports Sci Med. (2021) 20:86–93. doi: 10.52082/jssm.2021.86, PMID: 33707991 PMC7919351

[26] Yang Q. Strategies of movement education for the test of gross motor development in preschool children. J Xi’an Inst Phys Educ. (2017) 34:341–7. doi: 10.16063/j.cnki.issn1001-747x.2017.03.014

[27] Zhang Q. Experimental study on the effects of moderate and high intensity physical activity at different time on basic motor skills and physical health level of U6-7 Gannan Normal University (2021). doi: 10.27685/d.cnki.ggnsf.2021.000349

[28] Eyre ELJ Clark CCT Tallis J Hodson D Lowton-Smith S Nelson C . The effects of combined movement and storytelling intervention on motor skills in south Asian and white children aged 5–6 years living in the United Kingdom. Int J Environ Res Public Health. (2020) 17:3391. doi: 10.3390/ijerph17103391, PMID: 32414027 PMC7277335

[29] Magistro D Cooper SB Carlevaro F Marchetti I Magno F Bardaglio G . Two years of physically active mathematics lessons enhance cognitive function and gross motor skills in primary school children. Psychol Sport Exerc. (2022) 63:102254. doi: 10.1016/j.psychsport.2022.102254

[30] Xiao K. An empirical study on the effect of 8-week exercise intervention on the development of fundamental motor skills of 3 to 6 years old children (in Chinese). Nanjing Sport Institute (2022). doi: 10.27247/d.cnki.gnjtc.2022.000094

[31] Lu W. An experimental study on the effect of infant football on positive mental quality and physical health of preschool children from 5 to 6Years old. East China Normal University, (2022). doi: 10.27149/d.cnki.ghdsu.2022.001367

[32] Martin R Murtagh EM. An intervention to improve the physical activity levels of children: design and rationale of the ‘Active Classrooms’ cluster randomised controlled trial. Contemp Clin Trials. (2015) 41:180–91. doi: 10.1016/j.cct.2015.01.019, PMID: 25657052

[33] Routen AC Biddle SJ Bodicoat DH Cale L Clemes S Edwardson CL . Study design and protocol for a mixed methods evaluation of an intervention to reduce and break up sitting time in primary school classrooms in the UK: the CLASS PAL (physically active learning) Programme. BMJ Open. (2017) 7:e019428. doi: 10.1136//bmjopen-2017-019428 PMC5695437 29122808

[34] Page MJ McKenzie JE Bossuyt PM Boutron I Hoffmann TC Mulrow CD . The PRISMA 2020 statement: an updated guideline for reporting systematic reviews. Br Med J. (2021) 372:n71. doi: 10.1136/bmj.n71, PMID: 33782057 PMC8005924

[35] Higgins JPT Li T Deeks JJ. Chapter 6: choosing effect measures and computing estimates of effect In: Higgins JPT Thomas J Chandler J Cumpston M Li T Page MJ, editors. Cochrane handbook for systematic reviews of interventions version 6.1 (updated September 2020). Bristol UK: Cochrane (2020). 44–59.

[36] Higgins P Green S. Cochrane handbook for systematic reviews on interventions 5.3.3 user guide. (2014). Available at: www.cochrane-handbook.org

[37] The Nordic Cochrane Centre. Review manager; cochrane collaboration. London, UK: The Nordic Cochrane Centre (2014). 1–43.

[38] Deeks JJ Higgins J Altman DG. Analysing data and undertaking meta-analyses. Cochrane handbook for systematic reviews of interventions: Cochrane book series. USA: John Wiley & sons 20080:243–96.20.

[39] Yajnik CS Lubree HG Rege SS Naik SS Deshpande JA Deshpande SS . Adiposity and hyperinsulinemia in Indians are present at birth. J Clin Endocrinol Metab. (2002) 87:5575–80. doi: 10.1210/jc.2002-020434, PMID: 12466355

[40] Li B Liu J Ying B. Physical education interventions improve the fundamental movement skills in kindergarten: a systematic review and meta-analysis. Food Sci Technol. (2021) 42:e46721. doi: 10.1590/fst.46721, PMID: 39699478

[41] Yuxin Y Xiaofen L Wanxu L. Effects of early childhood physical activity program on gross motor development in preschool children: a meta-analysis. Chin J Evid Based Med. (2023) 23:319–26.

[42] He L Sheng Q. Physical Activity, Sedentary behaviour and sleep advice for the children of less than 5 years old. Digit Educ (in Chinese). (2020) 6:84–92.

[43] Gao W Wang H. Meta analysis of the effect of physical activity intervention on physical fitness in Chinese children aged 3-6 years. China School Health. (2021) 42:1311–1317+1322. doi: 10.16835/j.cnki.1000-9817.2021.09.009

[44] Utesch T Bardid F Büsch D Strauss B. The relationship between motor competence and physical fitness from early childhood to early adulthood: a meta-analysis. Sports Med. (2019) 49:541–51. doi: 10.1007/s40279-019-01068-y, PMID: 30747376

